# piRDisease v1.0: a manually curated database for piRNA associated diseases

**DOI:** 10.1093/database/baz052

**Published:** 2019-07-02

**Authors:** Azhar Muhammad, Ramay Waheed, Nauman Ali Khan, Hong Jiang, Xiaoyuan Song

**Affiliations:** 1 Hefei National Laboratory for Physical Sciences at the Microscale, CAS Key Laboratory of Brain Function and Disease, Neurodegenerative Disorder Research Center, School of Life Sciences, Division of Life Sciences and Medicine, University of Science and Technology of China, Hefei, Anhui, 230026, China; 2 Pattern Recognition and Information Retrieval lab, University of Science and Technology Beijing, Beijing 100083, China; 3 Key Laboratory of Wireless Optical Communication, Chinese Academy of Sciences, University of Science and Technology China, Hefei 230026, China; 4 Department of Biosciences, COMSATS University Islamabad, Sahiwal 57000, Pakistan

## Abstract

In recent years, researches focusing on PIWI-interacting RNAs (piRNAs) have increased rapidly. It has been revealed that piRNAs have strong association with a wide range of diseases; thus, it becomes very important to understand piRNAs’ role(s) in disease diagnosis, prognosis and assessment of treatment response. We searched more than 2500 articles using keywords, such as `PIWI-interacting RNAs’ and `piRNAs’, and further scrutinized the articles to collect piRNAs-disease association data. These data are highly complex and heterogeneous due to various types of piRNA idnetifiers (IDs) and different reference genome versions. We put considerable efforts into removing redundancy and anomalies and thus homogenized the data. Finally, we developed the piRDisease database, which incorporates experimentally supported data for piRNAs’ relationship with wide range of diseases. The *piRDisease* (*piRDisease* v1.0) is a novel, comprehensive and exclusive database resource, which provides 7939 manually curated associations of experimentally supported 4796 piRNAs involved in 28 diseases. *piRDisease* facilitates users by providing detailed information of the piRNA in respective disease, explored by experimental support, brief description, sequence and location information. Considering piRNAs’ role(s) in wide range of diseases, it is anticipated that huge amount of data would be produced in the near future. We thus offer a submitting page, on which users or researches can contribute in to update our *piRDisease* database.

## Introduction

PIWI-interacting RNAs (piRNAs) are a type of small non-coding RNAs, first described in germ cells, represented as one of the major group of small non-coding RNAs such as miRNA and siRNA (1). piRNAs play a crucial role to safeguard genome, maintain the genome complexity and integrity, as they suppress the insertional mutations caused by transposable elements. Previously, the role of piRNAs was confined to gonad development (2–4), whereas existing studies have revealed that the expression profile of piRNAs vary from central nervous system (brain) to colon, heart, kidney, liver, lung, small intestine, spleen, stomach, ovary and testis (5–9). Evidently, piRNAs play critical roles in disease progression, diagnosis and assessment of treatment response (9–18). Genome-wide profiling studies have revealed that the expression of piRNAs was dysregulated in various diseases. However, target based mechanistic studies revealed the regulatory role of piRNAs in various diseases (26, 31). piRNAs regulate target genes through base paring mechanism (19). For instance, piR-823 binds to HSF1 to promote its phosphorylation, which contributes to colorectal tumorigenesis (19). Knocking down of piR-34736 results in high expression of Bax/Bcl2 and repression of EMT-mediator Vimentin in head and neck cancer (20, 21). Accumlating evidences sugest that the change in expression of piRNAs and abrerration in target genes regulation will be potential diagnostic marker (18, 22–25). In recent years, a few databases have been developed to provide basic information related to piRNAs, such as piRNABank and piRBase, which provide comprehensive piRNA sequence and location information for several species (26, 28). piRNAQuest is another database resource, which offers a diverse narrative focusing on pseudogenes and synteny information including sequence and location data (27).

Several databases are available, which document non-coding RNAs such as long non-coding RNAs' and small non-coding RNAs' association with disease. These databases include LncRNADisease, Lnc2cancer, miR2Disease, miRCancer, circRNAdisease and Circ2Disease (28–33). However, there is no online database resource offering data on piRNAs and disease relationship. Therefore, we developed manually curated piRNA and disease association database resource, which provides experimentally supported piRNAs with their disease associations from literature.

## Construction and content

We searched PubMed for published research articles (34), using a list of keywords such as piRNAs, PIWI-interacting RNAs, PIWI-interacting RNAs involved in diseases and cancer, piRNAs and PIWI-interacting RNAs in diseases and cancers, respectively.

**Figure 1:**
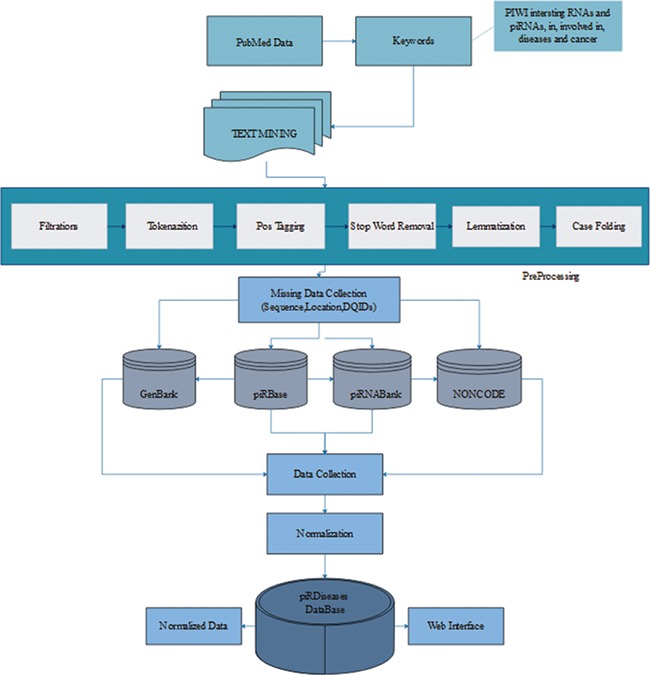
*piRDisease* database construction workflow. The *piRDisease* database was constructed from keywords search to data retrieval, preprocessing, normalization followed by adding missing data. Finally, all these data were stored in the *piRDisease* database.

We retrieved 2572 articles, filtered these articles on the basis of piRNAs’ disease associations to acquire more than 50 articles (Figure 1) (15, 23)**.** During data collection, we mainly focused on piRNAs’ association in respective diseases, illustrating their expression or mechanistic role in regulating target genes/proteins. Furthermore, we collected sequence and location information for those piRNAs, preceded by experimental methods, detail mechanism and description, *in vivo* or *in vitro* study, and the reference article’s PubMed identifier (ID) and title. After initial compilation of data, we observed that the data were in semantic form covering long textual strings including special characters, which usually creates problem during storage and retrieval of data from database. Therefore, before storing the data we applied several computational preprocessing methods, so that data can be curated smoothly (Figure 1).

We employed `Natural Language Toolkit’ and `TextBlob’ to apply natural language processing techniques on complex text data description (e.g. symbols, punctuation, double spaces, typo errors and long sentences etc.) extracted from literature particularly in two fields, such as `detailed mechanism’ and `description’ (39). Preprocessing involves several steps listed as follows.

**Tokenization:** The textual description of collected data from different research papers usually combines words and meaningless symbols e.g. special characters and punctuations. Such symbols create problems when we store the data in MYSQL. Tokenization filters out the meaningless symbols and divides the remaining text into tokens.

**Spell correction:** The unstructured attributes (e.g. detailed mechanism and description) of collected data may have spelling mistakes or typo errors. Therefore, we correct such mistakes in this step of preprocessing.

**Stop-word removal:** The text of a document often contains constructive terms (e.g. prepositions) and other language structures to connect sentences. Such terms are known as stop-words. We subtract stop-words from the preprocessed data.

**Word inflection and lemmatization:** Word inflection transforms words into their singular form and lemmatization shifts the comparative and superlative terms into their basic term. For example, inflection transforms the word `bugs’ into `bug’ and lemmatization shifts the word `computation’ into `compute’. We performed both word inflection and lemmatization to avoid the repetition of words that share the same basic term. Finally, we converted all the preprocessed words into lowercase (e.g. `Upregulated’ to `upregulated’).

After preprocessing, we categorized manually curated piRNAs’ disease association data in `annotation’ field based on experimental methods used in the reference studies. For example, piRNAs discovered from whole genome sequencing (WGS), RNA-Seq and microarray methods were denoted as `predicted’ **(**Table 1**)**. However, if piRNAs expression was quantitatively measured by RT-qPCR following these WGS experiments they were categorized as `related’. Finally, when piRNAs’ mechanistic (regulatory) role was elaborated by a series of experiments (e.g. knock-down, northern blotting, MTT assay, cell cycle analysis etc.), they were called as `validated’. In order to validate these records, data extraction from relevant genome version and reference databases was considered. We obtained piRNAs' missing sequence and location information from piRNAs reference databases (e.g. piRNABank and piRBase), and from other non-coding RNA databases (e.g. NONCODE 3.0).

**Table 1:** Annotation of piRNAs on the basis of experimental evidences

**Sr**	**Experiment methods in papers**	**Description**	**Annotation**	
1	Microarray, RNA-Seq	WGS	Predicted
2	Microarray, RNA-Seq, qPCR	WGS, RT-qPCR (expression validation)	Related
3	Northern blot, MTT assay, knock down, Western Blot, xenograft model etc.	Multiple experiments (mechanistic role validation)	Validated

After collection of the data, it was observed that data were highly diverse due to the complexity of nomenclature and various genome versions used by different non-coding RNAs databases in reference studies. piRNA-disease association studies incorporated data from various reference piRNA databases, and each of them has unique ID. For example, piRNABank and piRNAQuest use has_piR_000001 and piRBase follows piR_hsa_000001, which makes piRNA search quite challenging. However, DQ (accession ID) can be used to search exact piRNA in primary genome browsers such as GenBank as well as reference piRNA databases (26, 34, 35). Thus, we extracted DQ IDs for standardization, so that users can also use DQ ID data to search, explore and interpret results in *piRDisease* database **(**Figure 1**)**. Before storing data into our database, the data were normalized by removing data redundancy and anomalies.

**Figure 2:**
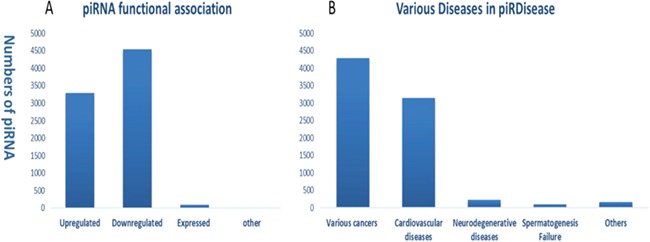
Statistics and distribution **(A)** of dysfunction types of piRNAs **(B)** in various disease types in *piRDisease* database.

**Figure 3:**
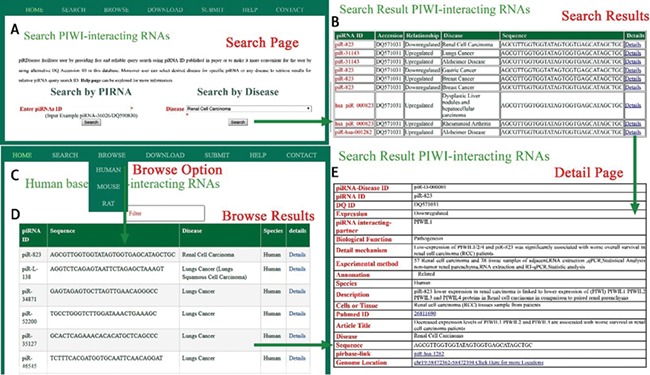
A schematic workflow for *piRDisease*. **(A)** Users can search individual piRNA or disease that is associated with piRNAs. **(B)** Searching results were shown. **(C, D)** piRNA-disease association data for three species were shown. **(E)***piRDisease* provided detailed information for relevant piRNA that was associated with specific disease.

Finally, all the mined data were stored in the form of database using MySQL (version 5.7.25). The web interface was built in HTML and CSS to make the web portal attractive. The data processing programs were written in PHP (5.7), ajax, JavaScript and the web services were built using Xamp server. The *piRDisease* database is freely available at http://piwirna2disease.org/.

In summary, *piRDisease* is a distinct database resource providing 7939 manually curated associations of experimentally supported 4796 piRNA involved in 28 different disease types.

## User interface

*piRDisease* provides `search’, `browse’ and `submit’ options on the home page. Users can search the database, entering piRNA ID or DQ-ID and select the specific disease or any disease to explore the piRNAs’ association (expression) in relevant disease, and this will display result page for searched piRNA’s (or disease associated piRNAs’) expression or interaction type in relevant disease (Figure 3). Currently users can browse piRNA-disease association data for three organisms (human, mouse and rat). The `submit’ button allows researchers to add in new data, which will be significant for updating information in *piRDisease* database. Further, users can click on `detailed page’ and it will reveal piRNA target genes, and detailed mechanism of piRNA expression or regulation of target genes. piRNAs are categorized as predicted, related and validated in annotation field in the database on the bases of experimental methods. Description provides the overall functional relationship followed by tissues or cells used in reference study (Figure 3). `Detailed page’ also provides piRNA sequence, location, species, PubMed IDs and title of the study. *piRDisease* uses `non fuzzy’ search so that exact match will be found. *piRDisease* also contains novel piRNAs as well as piRNA-like RNAs (piRNA-like) implicated in some diseases. *piRDisease* provides its own search ID for the piRNAs that do not have DQ IDs, piRNA-like and novel piRNAs.

## Utility and discussion

Evidently, piRNAs’ spatial and temporal expression is critical for normal cellular development and differentiation, ranging from embryonic stage to gonad development (7, 36–38). Hence, piRNAs dysregulated expression and peculiarly their target genes’ regulation can be a potentially diagnostic marker in wide range of diseases (7, 37, 38). Recent progression of studies enforced the role of piRNAs in various type of diseases, specifically different cancer types (Figures S1 and S2**)**. Enormous amount of piRNA-disease association data are expected to be produced in the near future. Hence, we developed *piRDisease* database by collecting piRNA-disease association data scattered in the literature. *piRDisease* is the first and novel piRNA database resource that contains 7939 piRNA-disease-associated entries, which comprises of 4796 unique piRNAs and 28 types of associated diseases in three species (human, mouse and rat; Figure 3, Table S1**)**. However, piRNAs involved in deep regulatory mechanism is still to be explored. For instance, when we search *piRDisease* with the search term `piR-651’, which is one of the highly explored piRNAs in various diseases in literature, we will retrieve eight results. We found that `piR-651’ is mostly upregulated in various cancer types such as breast cancer, gastric cancer, colon cancer, mesothelium, liver cancer and cervical cancer. However, only a few studies revealed detailed mechanistic roles of piR-651 in some diseases. For example, estrogen and androgen hormones treatment resulted in higher expression of piR-651 in prostate cancer. In addition, this piRNA overexpression was highly correlated with tumor propagation, which was mediated by cyclin D1 and CDK4 pathway in `non-small cell lung carcinoma’. These results suggested that `piR-651’ aberrant expression is significant to many cancer types, but only in a few cancer types its detailed mechanism was revealed. Currently, piRNA-disease association data are available for 28 diseases, of which 54% are various types of cancers; 40% are cardiovascular diseases; 4% are neurodegenerative diseases; and 1% are spermatogenesis-related and other diseases (Figure 2B).

## Conclusions

In order to provide biological community central resource to search, explore and investigate the piRNA-disease relationships, we developed *piRDisease* database, which is a convenient, comprehensive web-based database resource, providing detailed information about piRNAs’ role in various diseases.

*piRDisease* provides scientific community inclusive insights into piRNAs functional relationship in wide range of diseases. This novel and unique database resource will lead toward further research ideas.

## Future extension

Since piRNAs involved in diseases were explored vastly in the past few years, a huge amount of data is expected to be produced in the near future. We thus plan to update this data on yearly bases. In addition, we intend to build and incorporate some piRNA target prediction software based on some innovative algorithms.

## Authors Contribution

Mr Muhammad Azhar conceptualized idea, collected, stored and managed the data. Mr Muhammad Azhar and Mr Waheed Ramay contributed in building the database. Mr Nauman khan and Miss Hong Jiang cross checked the database. Dr Xiaoyuan Song supervised this work and manuscript.

## Supplementary Material

Supplementary_(figures_and_table)_file280319_baz052

## Notes

**Database URL**: http://www.piwirna2disease.org/index.php
