# Supplementary material for: piRDisease v1.0: a manually curated database for piRNA associated diseases
Source: Database (Oxford). 2019 Jul 2;2019:baz052. doi: 10.1093/database/baz052 (PMC6606758; doi:10.1093/database/baz052)
Supplement: Supplementary_(figures_and_table)_file280319_baz052 [file supplementary_(figures_and_table)_file280319_baz052.docx]

**Supplementary data file**

Web interface developed by using PHP,HTML,J Query to facilitate user

**Web interface**

**Figure S1:** Top 5 diseases in piRDisease database.

**Web interface developed by using PHP,HTML,J Query to facilitate user**

**Web interface**

**Figure S2**: Recently published papers with piRDisease association.

| **piRNA**  **Databases** | **Sequence** | **Location** | **Species** | **References** | **Diseases** |  |
| --- | --- | --- | --- | --- | --- | --- |
| piRNABank | Yes | Yes | Yes | Yes | No |  |
| piRNAQuest | Yes | Yes | Yes | No | No |  |
| piRBase | Yes | Yes | Yes | Yes | No |  |
| piRDisease | Yes | Yes | Yes | Yes | Yes |  |

**Table S1:** piRDisease compared with other databases. piRDisease is the only database which contains piRNAs association in various diseases.

**Upregulated**: In biological context, when cellular content is increased by quantity (protein or RNA) in response to external stimulus, metabolic changes, or disease condition, this process is called upregulation. Quantification of these changes can be measured by high-throughput sequencing methods at genome level or target RT-qPCR based amplification methods. In case of high-throughput sequencing (e.g., Microarray, small RNA-Seq) the rate of expression (e.g., Fold change, P value) is the measure of quantity of cellular component (protein or RNA) in a certain condition (disease) compared to normal samples. Consequently, if the rate of expression of (protein and RNA) is increased in a given condition, it is referred to as upregulation of protein or RNA content within cellular environment.

**Downregulated**: Similarly, in biological context, when cellular content is decreased by quantity (protein or RNA) in response to external stimulus, metabolic changes, or disease condition, this process is called downregulation. Quantification of these changes can be measured by high-throughput sequencing methods at genome level or target RT-qPCR based amplification methods. In case of high-throughput sequencing (e.g., Microarray, small RNA-Seq) the rate of expression (e.g., Fold change, P value) is the measure of quantity of cellular component (protein or RNA) in a certain condition (disease) compared to normal samples. Consequently, if the rate of expression of (protein and RNA) is decreased in a given condition, it is referred to as downregulation of protein or RNA content within cellular environment.

**Expressed**: Some studies measure expression of cellular content (RNA) by copy number rather than classic (fold change and P value) method. This method was adopted in some studies (25779424, 28289238) in piRNA-disease association data. So we observed that this term “express “is used by only two studies (25779424, 28289238) in *piRDisease* considering some key points in reference studies.

1. When referred content (piRNAs) “expressed” in certain number of patients from total number of patient samples and there copy number in terms of expression varies among these patients, compared to normal samples.
2. When (transcript) variants (SNP) to be quantified in number of patients.

**Others:** These terms include “biogenesis”, “Polymorphism” and “inversely correlated”, one entry for each. First two are biological mechanism, whereas in one study piRNA has antagonistic relationship with interleukin-4 (IL-4) via sequence complementary mechanism, so its described as “inversely correlated” in that paper.
